# Postoperative lymphocele causing obturator nerve entrapment, treated with percutaneous drainage and intranodal poppyseed oil (Lipiodol)-based lymphangiography

**DOI:** 10.1016/j.jvscit.2021.09.007

**Published:** 2021-10-02

**Authors:** Laurence Verhaeghe, Andries Van Holsbeeck, Joost Kager, Jozef Ampe, Koen Mermuys, Geert Maleux

**Affiliations:** a Department of Radiology, University Hospitals Leuven, Leuven, Belgium; b Department of Radiology, AZ Sint-Jan Bruges Hospitals, Bruges, Belgium; c Department of Urology, AZ Sint-Jan Bruges Hospitals, Bruges, Belgium

**Keywords:** Intranodal lymphangiography, Lipiodol, Lymphadenectomy, Obturator nerve entrapment, Postoperative lymphocele

## Abstract

Obturator nerve entrapment is a rare complication after pelvic surgery and is caused by a direct intraoperative injury or secondary to compression by a postoperative collection. We have presented the case of a 65-year-old man who had complained of right-sided medial groin pain 4 weeks after robot-assisted laparoscopic prostatectomy with bilateral pelvic lymphadenectomy. Pelvic magnetic resonance imaging showed bilateral lymphoceles with right-sided compression of the obturator nerve causing diffuse muscle edema in its innervation region. Percutaneous drainage and intranodal poppyseed oil (Lipiodol)-based lymphangiography led to a complete resolution of his symptoms.

Obturator nerve injury is a rare complication after robot-assisted laparoscopic prostatectomy with bilateral pelvic lymphadenectomy, with an incidence of 0.3%.^1^ Damage to the obturator nerve can result from an intraoperative injury, including stretching or transection of the nerve, or the patient's positioning, or can be secondary to compression by a postoperative collection. In the present report, we have described a postoperative lymphocele compressing the obturator nerve and causing acute denervation edema in the external obturator muscle and the adductor longus, brevis, and magnus muscle**,** for which successful percutaneous drainage and theranostic lymphangiography was performed.^2^ The patient provided written informed consent for the report of his case details and imaging studies.

## Case report

**Fig 1:**
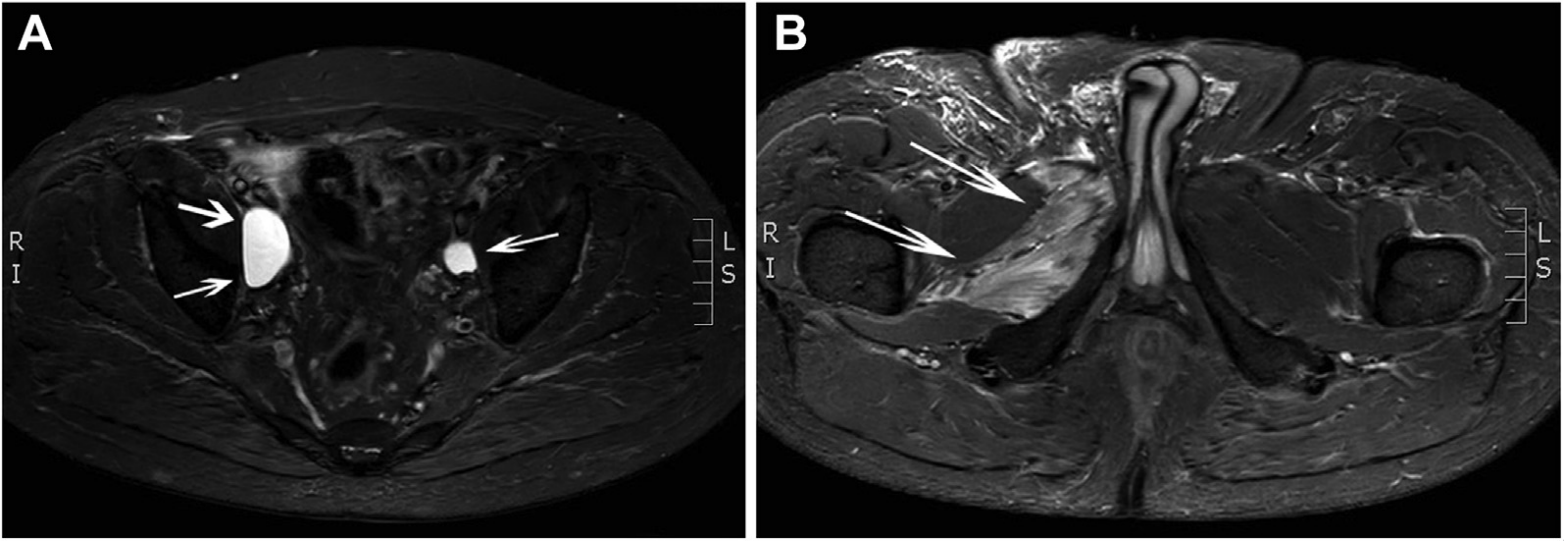
**A,** Axial short tau inversion recovery sequence showing bilateral postoperative fluid-containing collections (*arrows*). The largest collection was on the right and was located posteriorly to the common iliac vessels at the expected course of the obturator nerve (not directly visualized on this image). **B,** Axial short tau inversion recovery sequence showing a diffuse hyperintense signal in the external obturator muscle on the right representing edema (*arrow*).

A 65-year-old patient with a history of Gleason score 7 prostate adenocarcinoma underwent robot-assisted laparoscopic prostatectomy and bilateral pelvic lymphadenectomy. Four weeks later, he presented with right-sided medial thigh and groin pain with associated sensory loss in this region and weakness of the adductor muscles. Because of the clinical suspicion of obturator nerve compression, pelvic magnetic resonance imaging (MRI) was performed, demonstrating a bilateral intrapelvic fluid-containing collection (3.8 cm × 2.2 cm × 4.1 cm on the right and 1.6 cm × 1.5 cm × 2.6 cm on the left, both located posteriorly to the iliac vessels near the obturator canal), suggesting a lymphocele (Fig 1, *A*). Additionally, diffuse right-sided muscle edema in the external obturator muscle and the adductor longus, brevis, and magnus muscle was revealed (Fig 1, *B*). Because of the common innervation of these muscles by the obturator nerve, this pattern of edema was strongly suggestive for obturator nerve denervation with the lesion superiorly to the obturator foramen. No additional electromyography study was performed to confirm the MRI findings.

**Fig 2:**
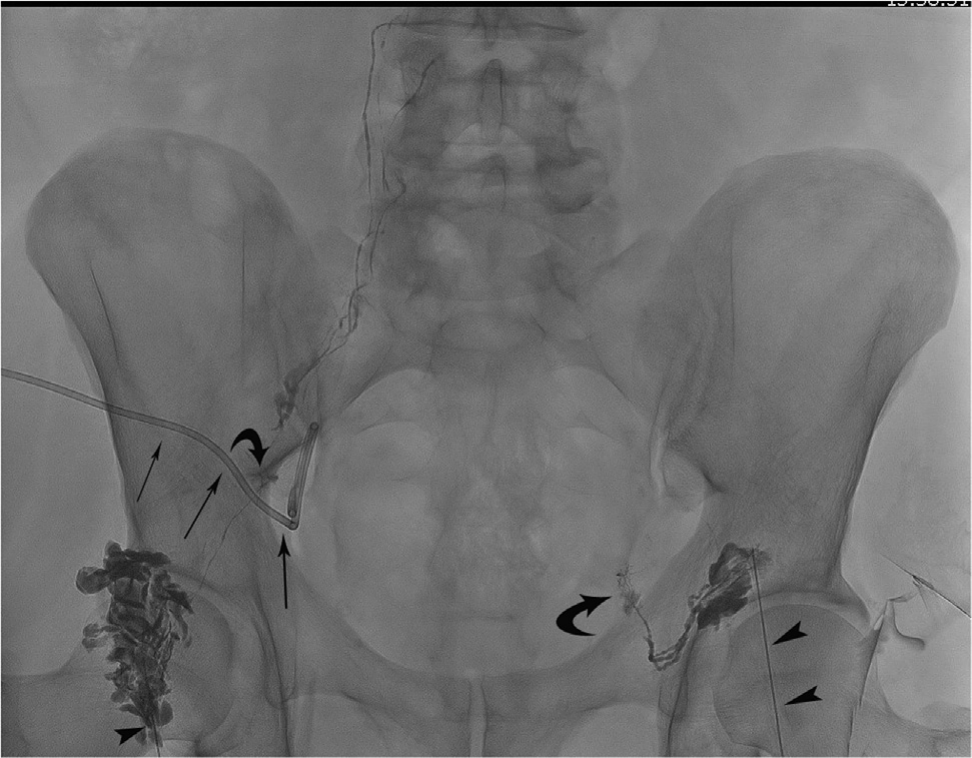
Fluoroscopic image during Lipiodol-based lymphangiography showing an intrapelvic drain (*thin arrows*) positioned in the lymphocele. Access to the lymphatic system was provided by a bilateral puncture of an inguinal lymph node (21-guage needle; *arrowheads*). A slow infusion of Lipiodol opacified the pelvic lymphatics, and a bilateral small lymphatic leakage (*curved arrows*) was revealed.

Treatment consisted of ultrasound-guided placement of a 7F pigtail catheter into the largest postoperative lymphocele on the right side and 15 mL of serous lymphatic fluid was extracted. Subsequently, bilateral inguinal intranodal lymphangiography was performed. An inguinal lymph node was punctured with a 21-guage needle (BD spinal needle; Becton Dickinson, Madrid, Spain) under ultrasound guidance and iodized poppyseed oil contrast medium (Lipiodol; Guerbet, Villepinte, France) was slowly inserted into the lymphatic system until the iliac and retroperitoneal lymphatic vessels were visualized. A total amount of 25 mL of Lipiodol was injected, and a small amount of lymphatic leakage was found on both sides (Fig 2). No complications related to the procedure developed. Two days later, the drain was extracted, and the patient was discharged from the hospital in good clinical health.

**Fig 3:**
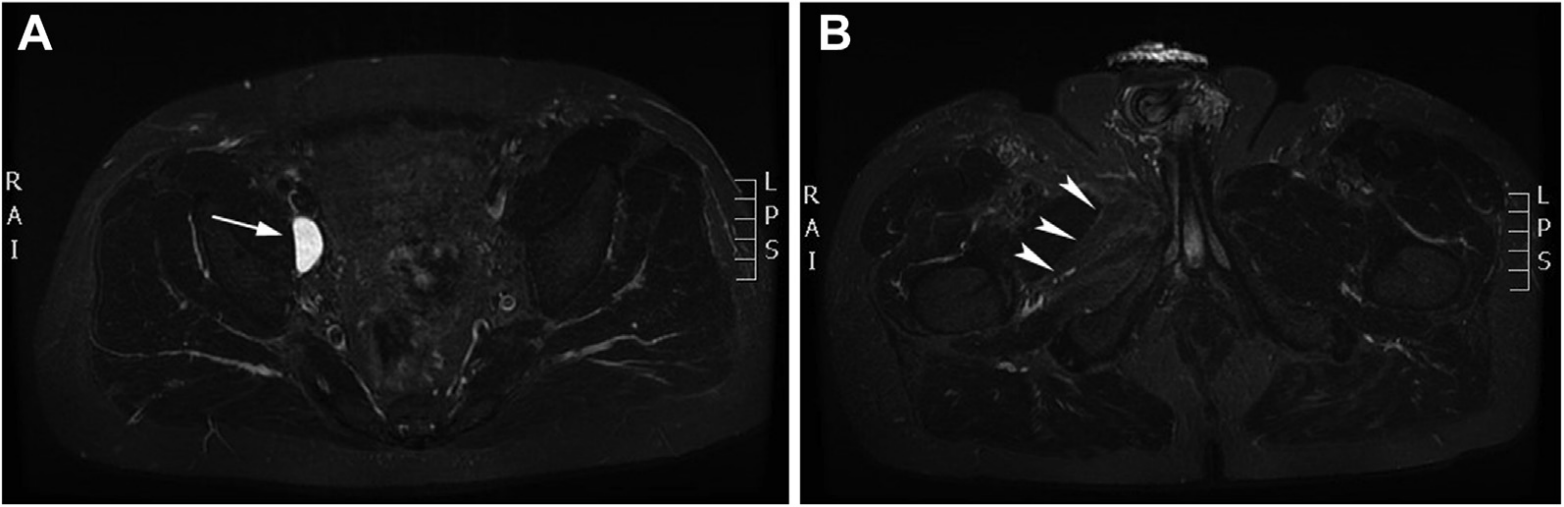
**A,** Axial short tau inversion recovery sequence after lymphangiography showing a small remnant of the lymphocele on the right (*white arrow*) and complete resolution of the lymphocele on the left. **B,** An obvious diminishment of the muscle edema (*arrowheads*) on the right can be visualized.

Three weeks later, both the medial groin pain and the associated sensory loss had diminished considerably. Follow-up MRI showed a significant decrease in volume of the lymphocele on the right side with a small remnant (2.9 cm × 1.3 cm × 3.0 cm) and complete resolution of the lymphocele on the left side. An obvious decrease had occurred in the diffuse muscle edema (Fig 3).

## Discussion

Most prostatectomies are accompanied by pelvic lymph node dissection (≤70% in 2010-2011; National Cancer Database study) to improve staging.^3^ The emergence of a lymphocele after lymph node resection is a common complication. Although most of these lymphoceles will remain subclinical, some will become symptomatic, predominantly owing to local compression. In the present case, the patient's symptoms had become clear 4 weeks after the initial surgery, which strongly argued against an intraoperative nerve injury and favored compression by a postoperative collection, including a hematoma or lymphocele, as demonstrated by MRI. In addition, treatment of the lymphocele was associated with disappearance of the postoperative symptoms, which would have been very unlikely if the nerve had been injured intraoperatively.

Because of its deep intrapelvic location, the obturator nerve is relatively protected, and obturator neuropathy is relatively uncommon. When it occurs, it is most frequently due to pelvic trauma or surgery and is related to mass effect and stretching, respectively.^4^ In the subacute reversible phase, nerve entrapment will cause edema of the denervated muscles with high signal intensity on MRI with fluid-sensitive sequences (T2-weighted short tau inversion recovery images).^2^

In the present case, the postoperative lymphocele on the right side was located near the obturator canal, at the expected course of the obturator nerve (not directly visualized on MRI) and was larger than the collection on the left side, resulting in unilateral symptoms. Together with the denervation edema in the external obturator muscle and adductor longus, magnus, and brevis muscle, this finding was highly suspicious for compression neuropathy of the obturator nerve caused by a postoperative lymphocele,^5^ in line with a more common cause of temporary loss of conduction (ie, nerve compression in locations where the nerve passes through narrow anatomic openings).

Percutaneous drainage (under ultrasound or computed tomography guidance) is often the initial management of symptomatic pelvic lymphoceles and in the present case, catheter drainage was associated with immediate control of the postoperative lymphocele. However, the recurrence rates have been as high as 25%, with a risk of infection of 50%.^6^ Open or laparoscopic marsupialization to connect the lymphocele with the peritoneal space is an effective treatment, with a success rate of 90%.^6^ However, a laparoscopic approach remains quite invasive.

Recently, bilateral intranodal Lipiodol-based lymphangiography has received a great amount of interest as a minimally invasive method to diagnose and treat lymphatic leakage and lymphoceles, with clinical success rates of 51% to 100% potentially related to a granulomatous or inflammatory reaction to Lipiodol that contributes to the therapeutic effect.^7^ In the present case, intranodal injection of Lipiodol confirmed the diagnosis of a lymphatic leak and led to a reduction or resolution of the lymphocele, with a subsequent significant decrease of muscle edema. Additional embolization with glue (a mixture of N-butyl cyanoacrylate and Lipiodol) can also be used and can increase the clinical success.^8^ Despite the combination of bilateral lymphadenectomy and bilateral intranodal Lipiodol-based lymphangiography, the patient did not develop postinterventional lower limb lymphedema, in line with previous reported results of bilateral intranodal lymphangiography for the treatment of postoperative chylous ascites^9^ and postoperative lymphatic leaks after robot-assisted laparoscopic pelvic resection.^10^ In contrast, pedal lymphangiography has been associated with peripheral lower limb edema related to disruption of the peripheral lymphatics as reported by Lee et al.^11^

## Conclusions

Obturator nerve entrapment caused by compression of a postoperative collection (hematoma or lymphocele) can result in a delayed presentation, making it difficult to differentiate between other causes of neurologic impairment. It is important to become familiar with this postoperative entity because early diagnosis and treatment can prevent irreversible neurologic damage. Percutaneous drainage, followed by intranodal Lipiodol-based lymphangiography, can be a successful treatment that is less invasive than laparoscopic marsupialization.
